# Neurophysin-I dynamics upon different pituitary provocation tests in healthy participants

**DOI:** 10.1530/EC-25-0929

**Published:** 2026-05-11

**Authors:** Andi Nikaj, Cihan Atila, Deborah Rudin, Dino Luethi, Clara O Sailer, Bettina Winzeler, Sandrine A Urwyler, Julie Refardt, Matthias E Liechti, Mirjam Christ-Crain

**Affiliations:** ^1^ Department of Endocrinology, Diabetology and Metabolism, University Hospital Basel, Basel, Switzerland; ^2^ Department of Clinical Research, University Hospital Basel, University of Basel, Basel, Switzerland; ^3^ Division of Clinical Pharmacology and Toxicology, Department of Pharmaceutical Sciences, University of Basel, Basel, Switzerland; ^4^ Division of Clinical Pharmacology and Toxicology, Department of Biomedicine, University Hospital Basel, Basel, Switzerland; ^5^ Division of Endocrinology, Diabetes and Hypertension, Department of Medicine, Brigham and Women’s Hospital, Harvard Medical School, Boston, Massachusetts, USA; ^6^ Department of Endocrinology, Diabetology and Clinical Nutrition, University Hospital Zurich, Zurich, Switzerland; ^7^ Department of Internal Medicine, Section of Endocrinology, Erasmus Medical Center, Rotterdam, The Netherlands

**Keywords:** oxytocin, diabetes insipidus, AVP-deficiency, hypertonic saline, arginine, macimorelin, glucagon, posterior pituitary, neurophysin

## Abstract

**Background:**

Oxytocin (OXT) is known for its role in regulating social behavior, such as emotion recognition and bonding. The evidence for clinically relevant OXT deficiency is constantly increasing, but the measurement of OXT is challenging. In contrast, neurophysin-I (NP-I), the carrier protein of OXT and co-released equimolarly, is a stable surrogate biomarker for OXT. This study evaluated OXT and NP-I levels upon four different pituitary provocation tests.

**Design:**

Secondary analysis of four interventional diagnostic trials at the University Hospital Basel, Switzerland.

**Methods:**

Sixty-six participants underwent a pituitary provocation test, 11 the hypertonic saline test, 20 the arginine infusion test, 25 the oral macimorelin test, and 10 the glucagon injection test. Plasma NP-I and OXT were measured at baseline and once the plasma sodium level reached ≥150 mmol/L for the hypertonic saline, after 60 min for the arginine infusion, after 60, 120, and 180 min for the glucagon injection, and after 45 min for the oral macimorelin test. The primary outcome was change from baseline to maximally stimulated NP-I levels.

**Results:**

Median (IQR) NP-I plasma levels increased minimally in response to hypertonic saline (baseline: 55 pM, stimulated: 59 pM, relative median change of −8% (−16, +9)) and significantly to glucagon injection (baseline: 82 pM, stimulated: 94 pM, relative median change of +18% (+7, +28)). Median OXT levels showed similar trends in response to hypertonic saline (baseline: 0.36 pg/mL (0.27, 0.45), stimulated: 0.48 pg/mL (0.43, 0.59), relative median change of +22% (−15, +96)) and to glucagon injection (baseline: 82.7 pg/mL (62.3, 94.3), stimulated: 93.4 pg/mL (87.2, 121), relative median change of +25% (+6, +59). Neither the NP-I plasma levels nor OXT levels increased upon arginine infusion and oral macimorelin.

**Conclusion:**

NP-I showed minimal responses to pituitary provocation tests, paralleling OXT dynamics. These findings highlight NP-I’s potential as a reliable and technically robust surrogate biomarker for OXT activity, warranting further evaluation in clinical and experimental settings.

## Introduction

Oxytocin (OXT) is known for its role in regulating social behavior such as emotion recognition, empathy, and social bonding (1, 2). The evidence for clinically relevant OXT deficiency is increasing, as is the interest in accurate and reliable measurement methods of OXT (3, 4). A recent diagnostic approach using 3,4-methylenedioxymethamphetamine (MDMA) as an OXT stimulator has revealed concomitant OXT deficiency in patients with arginine vasopressin (AVP) deficiency (5). However, progress in developing MDMA-based or alternative stimulation tests has been limited by the methodological challenges of measuring endogenous OXT. Widely used competitive immunoassays are limited by nonspecific antibody binding, matrix effects, and poor reproducibility (6, 7). Moreover, OXT has a short half-life and is unstable, making measurements difficult to interpret in non- or weakly stimulated conditions (8).

In contrast, neurophysin-I (NP-I), the carrier protein of OXT, co-released equimolarly, exhibits high stability and can be quantified via sandwich immunoassays (6). Across conditions known to stimulate OXT release, NP-I closely mirrors changes in circulating OXT dynamics. Strong correlations between OXT and NP-I have been observed during pharmacological stimulation with MDMA (*R* = 0.92, *R* = 0.82) and in naturally increased states such as late pregnancy (*R* = 0.84) (9, 10, 11). These findings indicate that under stimulated conditions, NP-I reliably reflects endogenous OXT dynamics and may serve as a robust surrogate biomarker.

The dynamics of NP-I during other established pituitary provocation tests have not been investigated to date. Previous studies assessing OXT responses upon hypertonic saline, arginine infusion, and oral macimorelin found that only hypertonic saline induced a rise in OXT levels (50%), although responses were highly variable, whereas arginine and macimorelin showed no significant effect (12). Similarly, glucagon injection provocation produced only minor, clinically irrelevant changes in OXT levels (13). More recently, administration of kisspeptin (19%) or melatonin (26%) produced modest increases of comparable magnitude (14, 15). It is uncertain whether prior studies missed true stimulatory responses because of assay limitations or whether these stimuli indeed fail to provoke measurable OXT release.

Therefore, this study aimed to evaluate the NP-I dynamics in response to hypertonic saline, arginine, macimorelin, and glucagon provocation in healthy volunteers and compare the results with OXT dynamics. We hypothesized that NP-I, unlike OXT, might show a clinically relevant response in healthy controls.

## Materials and methods

### Trial design

This is a secondary analysis of four diagnostic interventional studies conducted in healthy adults at the University Hospital Basel. The present secondary analysis included 11 healthy participants undergoing hypertonic saline provocation (out of the original 20 due to missing samples) (study 1), 20 healthy participants undergoing an arginine provocation (study 2), 25 healthy participants undergoing an oral macimorelin provocation (study 3), and 10 healthy participants undergoing a glucagon injection provocation (study 4). Full details of the studies’ rationale, design, and statistical analysis have been published elsewhere (16, 17, 18, 19).

### Participants

Individuals were excluded if they had AVP deficiency or resistance (central or nephrogenic diabetes insipidus), primary polydipsia, hypo- or hypernatremia, chronic or treatment-dependent illnesses, chronic alcohol use, a BMI outside 18–28 kg/m^2^ (>25 kg/m^2^ for the glucagon protocol), use of medications other than oral contraceptives, or if they were pregnant or breastfeeding.

### Hypertonic saline infusion protocol

A standardized hypertonic saline infusion test was conducted as described previously (16). Briefly, participants arrived in the morning after an overnight fast (≥8 h without food, ≥2 h without fluids) and refrained from smoking and alcohol for at least 24 h. Two antecubital venous catheters were placed for infusion and blood sampling. After a 30-min supine rest, baseline clinical assessments and blood samples were obtained to measure plasma sodium, copeptin, OXT, and NP-I. The test commenced with a 250-mL bolus of 3% saline infused over 15 min, followed by continuous infusion at 0.15 mL/kg/min. Plasma sodium was monitored at 30-min intervals, and infusion was stopped once concentrations exceeded 150 mmol/L – a level demonstrated to produce robust copeptin stimulation with acceptable side effects. At this endpoint, clinical parameters were reassessed and blood was collected for stimulated plasma copeptin, OXT, and NP-I determinations.

### Arginine infusion protocol

A standardized arginine infusion provocation test was performed as described previously (17). Briefly, participants arrived in the morning after an overnight fast (≥8 h without food and ≥2 h without fluids) and refrained from smoking and alcohol for at least 24 h. An antecubital venous catheter was inserted, followed by a 30-min rest in the supine position. Baseline clinical parameters and blood samples were collected for measurement of plasma copeptin, OXT, and NP-I. Arginine (L-arginine-hydrochloride 21%; Braun, B. Braun Melsungen AG) was administered at 0.5 g/kg body weight, diluted in 500 mL of 0.9% saline, and infused over 30 min. At 60 minutes – the expected peak of arginine stimulation–clinical parameters were reassessed, and blood was drawn for determination of stimulated plasma copeptin, OXT, and NP-I concentrations.

### Oral macimorelin protocol

A standardized oral macimorelin stimulation test was performed as described previously (18). Briefly, participants arrived in the morning following an overnight fast of at least 8 h without food or fluid intake, refrained from smoking on the day of testing, and avoided physical activity for 24 h beforehand. Baseline clinical parameters and blood samples were obtained for measurement of plasma copeptin, OXT, and NP-I immediately before ingestion of macimorelin dissolved in water at a dose of 0.75 mg/kg body weight, administered over 10 min. At 45 minutes – corresponding to the expected peak growth hormone response – clinical parameters were reassessed, and blood samples were collected for analysis of stimulated copeptin, OXT, and NP-I levels.

### Glucagon injection protocol

A standardized glucagon stimulation protocol was performed as described previously (19). Briefly, participants arrived in the morning after an overnight fast of at least 8 h without food or fluids. After 30 min in a semi-recumbent resting position, a venous catheter was inserted. To mitigate nausea, ondansetron 8 mg was administered orally 10 min before the start of the test. Baseline blood sampling was performed, followed by subcutaneous injection of either glucagon 1 mg (Glucagen® NovoNordisk – Hypokit) or placebo (1 mL 0.9% sodium chloride). Blood samples were collected at baseline and at 60, 120, and 180 min for measurement of plasma copeptin, OXT, and NP-I. Each participant completed two study days – one with glucagon and one with placebo – administered in randomized order with a minimum washout of 48 h. Both participants and study personnel were blinded to the treatment. Only glucagon-day data were used for the present analyses.

### Laboratory measurements

Blood samples for plasma OXT and NP-I analysis were taken in lithium heparin and EDTA tubes, immediately centrifuged at 4°C at 3,000 rpm for 10 min, and stored at −80°C until central batch analysis. For the hypertonic saline and arginine infusion tests, OXT concentrations were determined using a radioimmunoassay (RIA) on lithium heparin plasma performed in duplicate after acetone–ether extraction with the Pitt-Ab-2 antibody and HPLC-purified [^125^I]-oxytocin. The standard curve was linear between 0.25 pg/mL and 5.0 pg/mL, and the limit of detection was 0.2 pg/mL. The intra-assay and inter-assay coefficients of variation were 4.2 and 12.4%, respectively. The antiserum exhibited cross-reactivity with arginine vasotocin, but <1% with arginine vasopressin, lysine vasopressin, or desmopressin. For the macimorelin and glucagon provocation tests, plasma OXT was measured using a commercial ELISA kit (ENZO Life Sciences, USA) on EDTA plasma, following the manufacturer’s protocol. Plasma samples were diluted in TFA–H_2_O, and the collected supernatant was loaded onto an Oasis® PRiME HLB 96-well plate (30 mg sorbent, Waters Corporation, USA). Samples were eluted with 95% acetonitrile +5% TFA–H_2_O (0.1%), reconstituted, and transferred to a goat anti-rabbit IgG microtiter plate. After overnight incubation with the oxytocin conjugate, plates were read at 405 nm, and concentrations were calculated using a four-parameter logistic curve fit. The sensitivity of the assay was 15 pg/mL (range: 15.6–1,000 pg/mL). The intra-assay and inter-assay coefficients of variation were 1.6 and 5.0%, respectively. According to the manufacturer, the antiserum displayed cross-reactivity of 7% with mesotocin, 7.5% with arginine vasotocin, and <0.02% with other related peptides.

NP-I was quantified by using the Oxytocin-Neurophysin 1 Prepropeptide *in vitro* SimpleStep sandwich ELISA immunoassay kit (Abcam, UK) according to the manufacturer’s protocol, with a sensitivity of 10 pg/mL and a detection range of 125–8,000 pg/mL. Unextracted plasma EDTA samples were diluted 1:4 in assay buffer for analysis. The CV for dilution-corrected concentrations demonstrated excellent reproducibility, with an inter-assay CV of 10.0% for plasma samples and an intra-assay CV of 3.2%. The required sample volume per NP-I measurement was 50 μL. We performed conversions to express the concentrations of NP-I in molar units, standardizing the measurement based on the number of molecules per unit volume. The factor for converting NP-I in pg/mL to pM is ∼0.1. Raw data in pg/mL are presented in the supplementary information (Table S1 (see section on the Supplementary materials given at the end of the article)).

### Statistical analysis

Demographic information was described using median (IQR) or absolute (relative) frequency, as appropriate. Summary statistics for all outcomes at each measurement time are provided for each provocation test separately.

The primary objective of this analysis was to evaluate the maximum change in plasma NP-I and OXT levels for each pituitary provocation test. Differences (baseline to expected peak copeptin provocation) were assessed using Wilcoxon’s signed-rank test. For glucagon provocation, the highest value recorded during the entire session was used as the maximally stimulated value. Relative changes were calculated for each participant. Median and interquartile range (IQR) of these values were reported. A percent change of 0% refers to the same basal and stimulated level; values above 0% refer to an increase in stimulated hormone levels, and values below 0% refer to a decrease in stimulated hormone levels in reference to the basal level. For this exploratory design and given the lack of data in humans, the sample size was not calculated upfront. Statistical analyses were performed using R version 4.4.1. Confirmatory analyses were restricted to the primary outcome in healthy volunteers. We tested the null hypothesis that baseline and maximal NP-I levels did not differ across the various provocation tests using a two-sided Wilcoxon signed-rank test for paired samples, with a significance level of *α* = 0.05. Hypothesis testing was two-sided, and *P*-values <0.05 were considered statistically significant. All further analyses are exploratory without hypothesis testing. Although this study is exploratory and not formally powered *a priori*, we conducted a post hoc sensitivity analysis to contextualize the detectable effect size. Based on the observed variability of percent change, a sample size of approximately 10 participants provides ∼80% power (two-sided *α* = 0.05) to detect a within-subject change of approximately 17–20% in NP-I following arginine or macimorelin.

## Results

### Baseline characteristics

Of the 66 healthy volunteers included in this study, the median (IQR) age was 24 (22, 27) years, 35 (53%) were female, and the median BMI was 22.3 kg/m^2^ (20.4, 24.3). Baseline characteristics of participants undergoing each pituitary provocation test are presented in Table 1.

**Table 1:** Baseline characteristics.

	Hypertonic saline infusion	Arginine infusion	Oral macimorelin	Glucagon injection
Participants, *n*	11	20	25	10
Age, years	24 (23, 28)	27 (24, 29)	22 (22, 24)	25 (23, 28)
Sex, female	7 (64%)	10 (50%)	12 (48%)	6 (60%)
BMI, kg/m^2^	23.8 (21, 24.9)	20.6 (19.8, 20.8)	22.3 (21.5, 24.4)	22.6 (20.6, 24.5)

Data presented as median (IQR) or frequency (%). BMI, body mass index.

### NP-I and OXT upon hypertonic saline infusion

**Figure 1:**
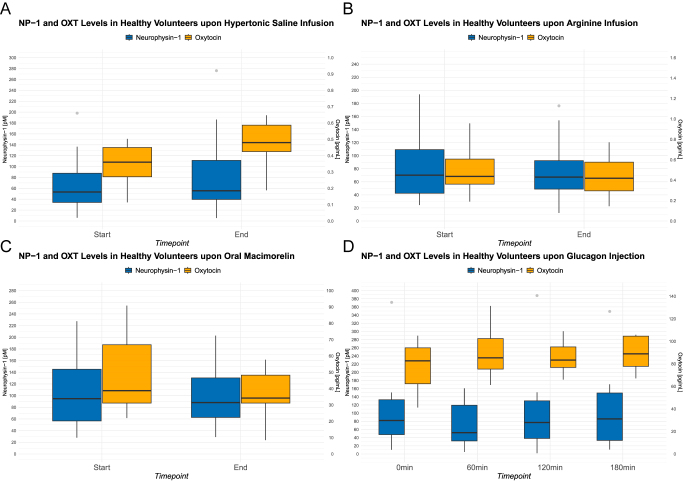
Time course of NP-I and OXT upon hypertonic saline (A), arginine (B), macimorelin, (C) and glucagon (D). Boxes span the IQR; the thick horizontal line is the median. Whiskers indicate the extreme values lying within the box edge and 1.5 times the IQR. All other values in gray are considered to be outliers. Outliers that exceeded the displayed *y*-axis limits are not shown but were retained in all analyses.

The time course of plasma NP-I upon hypertonic saline in healthy volunteers is presented in Fig. 1A and Table 2. The median time until serum sodium ≥150 mmol/L was 120 min (90, 150). The plasma NP-I level at baseline was 55 pM (35, 167). Upon provocation, plasma NP-I level was 59 pM (41, 151) (*P* = 0.28), consistent with a median percent change of −8% (−16, +9). Calculated dynamics of plasma OXT concentrations (based on a reduced sample set compared to Sailer *et al.*) (12) showed a non-significant increase in the OXT concentration (basal plasma OXT: 0.36 pg/mL (0.27, 0.45); stimulated plasma OXT: 0.48 pg/mL (0.43, 0.59); *P* = 0.24), corresponding to a median percent change of +22% (−15, +96).

**Table 2:** Laboratory results of NP-I and OXT for each provocation test.

Provocation	Hypertonic saline infusion	Arginine infusion	Oral macimorelin	Glucagon injection
Neurophysin-I (pM)				
Baseline	55 (35, 167)	70 (42, 109)	95 (57, 145)	82 (47, 133)
Stimulated	59 (41, 150)	68 (49, 103)	89 (65, 137)	94 (38, 161)
Relative median change (%)	−8 (−16, +9)	+0.3 (−13, +15)	+2 (−15, +11)	+18 (+7, +28)
*P*-value	0.28	0.73	0.65	0.01
Oxytocin (pg/mL)				
Baseline	0.36 (0.27, 0.45)	0.44 (0.36, 0.6)	38.7 (31, 67)	82.7 (62, 94)
Stimulated	0.48 (0.43, 0.59)	0.42 (0.3, 0.58)	34.2 (31, 48)	93.3 (38, 161)
Relative median change (%)	+22 (−15, +96)	−11 (−34, +29)	−5 (−32, +10)	+25 (+6, +59)
*P*-value	0.24	0.26	0.2	0.01

Data presented as median (IQR). Conversion factors for NP-I: 1 pg/mL ≈ 0.1 pM for NP-I.

### NP-I and OXT upon arginine infusion

The time course of plasma NP-I upon arginine in healthy volunteers is presented in Fig. 1B and Table 2. The plasma NP-I level at baseline was 70 pM (42, 109). Upon provocation, the plasma NP-I level was 68 pM (49, 103) (*P* = 0.73), consistent with a median percent change of +0.3% (−13, +15). Plasma OXT concentrations remained unchanged (basal plasma OXT: 0.44 pg/mL (0.36, 0.6), stimulated plasma OXT: 0.42 pg/mL (0.3, 0.58), *P* = 0.26), consistent with a median percent change of −11% (−34, +29).

### NP-I and OXT upon oral macimorelin

The time course of plasma NP-I upon macimorelin in healthy volunteers is presented in Fig. 1C and Table 2. The plasma NP-I level at baseline was 95 pM (57, 145). Upon provocation, the plasma NP-I level was 89 pM (65, 137) (*P* = 0.65), consistent with a median percent change of +2% (−15, +11). Plasma OXT remained unchanged in response to oral macimorelin test (basal plasma OXT: 38.7 pg/mL (31, 67); stimulated plasma OXT: 34.2 pg/mL (31, 48); *P* = 0.2), consistent with a median percent change of −5% (−32, +10).

### NP-I and OXT upon glucagon injection

The time course of plasma NP-I upon glucagon in healthy volunteers is presented in Fig. 1D and Table 2. The plasma NP-I level at baseline was 82 pM (47, 133). Upon glucagon provocation, the plasma NP-I level increased significantly to 94 pM (38, 161) (*P* = 0.01), consistent with a median percent change of 18% (+7, +28). Plasma OXT concentrations revealed a significant increase in OXT concentrations (basal plasma OXT: 82.7 pg/mL (62.3, 94.3); stimulated plasma OXT: 93.4 pg/mL (87.2, 121); *P* = 0.01), consistent with a median percent change of +25% (+6, +59). At baseline, the glucose level was 5.0 mmol/L (4.6, 5.2). Following the glucagon injection, glucose levels peaked at 8.1 mmol/L (7.2, 9.4) after 30 min and then decreased to a low-normal level of 3.8 mmol/L (3.5, 4.5) after 120 min.

## Discussion

The main finding of the present study is that NP-I levels did not increase upon arginine or macimorelin provocation and showed minor and variable increases upon hypertonic saline and glucagon provocation. This response pattern parallels that observed for OXT under the same provocation conditions. Accordingly, our results confirm that NP-I is a stable surrogate biomarker for OXT, given its matching provocation profile.

Reliable assessments of endogenous OXT are crucial for understanding its role in human physiology and pathology (2). Despite ongoing research, direct OXT measurements remain technically challenging (20). Methods in use, such as RIA or ELISA, have several weaknesses affecting sensitivity and specificity (21). Cross-reactivity with structurally similar peptides and the need for complex sample preparation limit the reliability of these approaches (21). Liquid chromatography–mass spectrometry offers superior specificity, but is costly and technically demanding, restricting its broader use and accessibility (22). NP-I comprises 94 amino acids and is therefore more than ten times bigger than OXT. Unlike OXT, this size difference allows NP-I to be measured using a sandwich immunoassay, which employs paired capture and detection antibodies that bind to non-overlapping epitopes (10). Additionally, its longer half-life has an impact on the measurement accuracy and may allow the detection of subtle changes. Nevertheless, our results show that, similar to OXT, NP-I does not exhibit clinically relevant changes after arginine, macimorelin, or hypertonic saline. The modest rise observed after glucagon (ΔNP-I = 12 pM) was smaller than that seen with strong OXT-releasing stimuli such as MDMA (ΔNP-I ≈ 1,443 pM) (9), suggesting a physiologically weak or indirect stimulus. Glucagon causes transient hyperglycemia followed by a rapid fall in glucose, and relative hypoglycemia is known to activate both anterior pituitary hormones and AVP release from the posterior pituitary (23, 24). Previous studies have shown that insulin-induced hypoglycemia can increase OXT release, supporting the notion that the glucagon-related NP-I rise likely reflects secondary metabolic effects rather than direct stimulation of magnocellular OXT neurons (25, 26).

Unfortunately, correlation analyses between NP-1 and OXT were not feasible due to methodological heterogeneity across studies (OXT measurement were done with RIA vs ELISA), combined with the instability and short half-life of OXT, which makes a direct comparison in our data difficult, especially for levels in the low-normal range.

Thus, our findings underline that a strong stimulus such as MDMA is needed to demonstrate the correlation of OXT and NP-I (9). With increasing recognition of a clinically relevant OXT deficiency, there is a pressing need for more reliable diagnostic tools (27, 28, 29, 30). A stimulation test is considered clinically useful when stimulated values rise consistently and reproducibly ≥100% compared to baseline variability and assay error. This threshold reflects the principle that diagnostically meaningful separation requires changes substantially exceeding biological variability and analytical imprecision, rather than moderate relative increases in the range of 20–30%, which may not necessarily reflect robust diagnostic separation. From a statistical perspective, effect sizes approaching at least two standard deviations are typically required to achieve acceptable discrimination (i.e. sensitivity and specificity in the range of ∼80–90%), thereby ensuring that the observed increase is biologically robust and clinically interpretable (31). To date, MDMA is the only stimulus that robustly increases both OXT and NP-I, and a second independent study has recently confirmed the near-perfect correlation between OXT and NP-I under stimulation (5, 9). Future studies should either investigate a feasible MDMA-based stimulation test for clinical practice or focus on other possible stimulation tests, such as estrogen (14, 32).

Some limitations should be considered for our study. First, this is a secondary analysis of four prospective diagnostic studies. Despite careful blood sampling, immediate centrifugation, and quick storage at −80 °C, a certain decay of NP-I cannot be excluded. Second, the sample size was limited and did not allow subgroup analyses. Third, data on repeated freeze–thaw cycles for NP-I are lacking. We tried to minimize variability by using previously unthawed samples in most cases (>70%; *n* = 36 samples, 2 cycles, −80°C). Fourth, OXT was measured using two different assays across protocols, and the absence of split-sample measurements precluded formal method-comparison analyses to exclude potential assay-related bias in the association between NP-I and OXT. Strengths of our study include the use of multiple well-established provocation tests, and a technically robust NP-I assay with high analytical specificity. By directly comparing NP-I and OXT responses, the study provides valuable insights into the future use of NP-I as a surrogate biomarker of OXT.

In summary, NP-I did not increase upon arginine infusion and oral macimorelin but showed small increases upon hypertonic saline infusion and glucagon injection, similar to what has been shown for OXT levels (12, 13). Our findings thus did not reveal any missed stimulatory responses upon different pituitary stimuli and further that NP-I may act as a reliable surrogate biomarker for OXT. Future research should systematically evaluate NP-I responses to various OXT-releasing stimuli across different biological matrices to establish its utility in clinical practice and research.

## Supplementary materials





## Declaration of interest

MEL is a consultant for Mind Medicine Inc. and Lykos Therapeutics. All other authors declare no competing interests.

## Funding

MCC received a grant from the Swiss National Science Foundation (32473B_162608). AN received an individual research grant from the Swiss National Science Foundation. CA and COS both received the Young Talents in Clinical Research grant of the Swiss Academy of Medical Sciences and G & J Bangerter-Rhyner Foundation.

## Author contribution statement

AN contributed to data collection, did the data analysis, did the data interpretation, did the literature search, and wrote and edited the manuscript. CA, SU, BW, and JR wrote one of the protocols, contributed to data collection, and edited the manuscript. DR and DL performed the measurements and edited the manuscript. COS edited the protocols, contributed to data collection, and edited the manuscript. MCC and MEL contributed to data analysis and data interpretation, edited the manuscript, and supervised all steps of the conduct of the study.

## Ethics statement

The studies conformed to the Declaration of Helsinki and were approved by the Ethics Committee of Northwest Switzerland. All four studies were preregistered on ClinicalTrials.gov (NCT02647736, NCT01879137, NCT03844217, NCT04550520). Written informed consent was obtained from each participant after a full explanation of the purpose and nature of all procedures.
