# Supplementary material for: Neurophysin-I dynamics upon different pituitary provocation tests in healthy participants
Source: Endocr Connect. 2026 May 11;15(5):e250929. doi: 10.1530/EC-25-0929 (PMC13188200; doi:10.1530/EC-25-0929)
Supplement: Supplementary file 1 [file supplementary_figure.pdf]

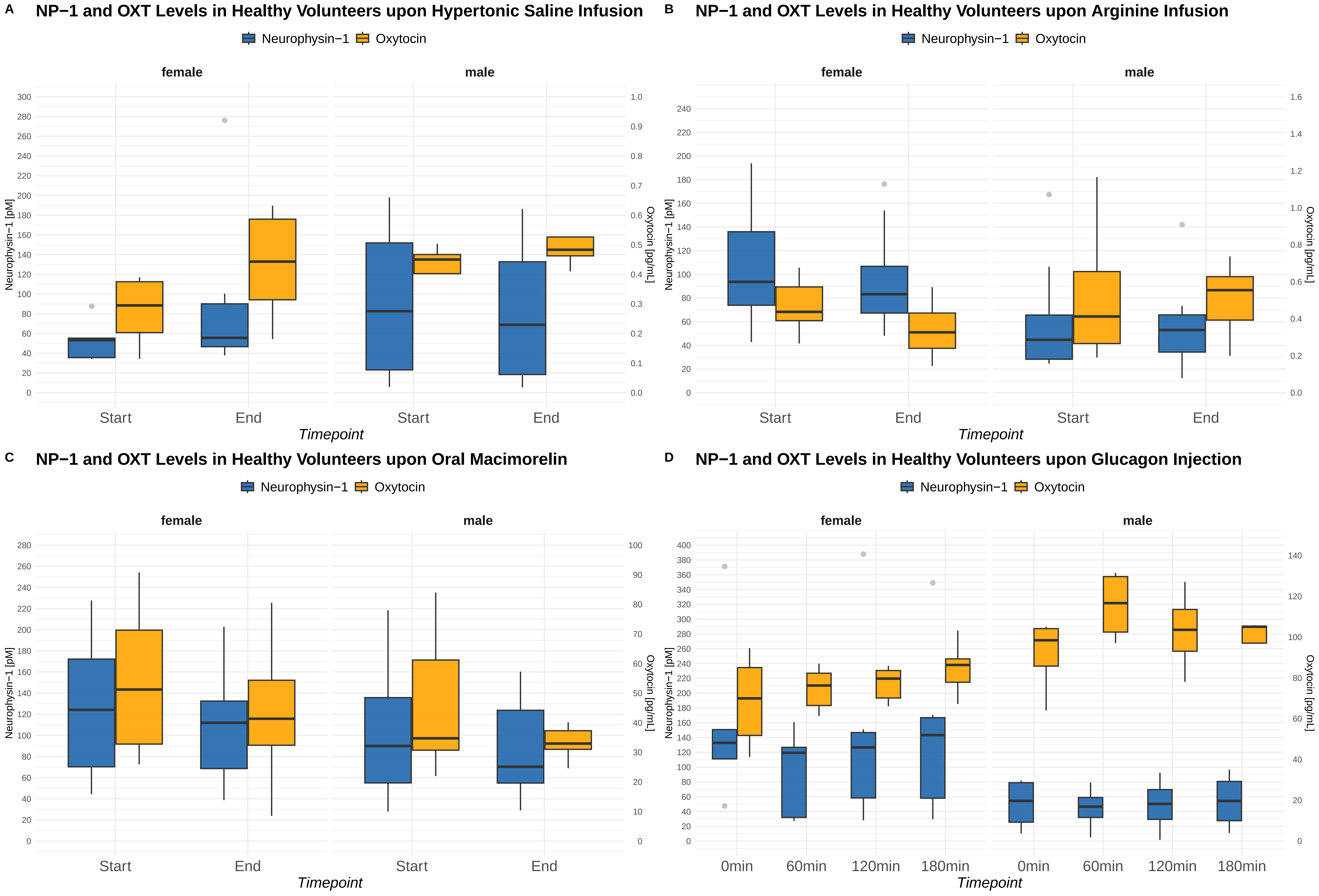
A
NP−1 and OXT Levels in Healthy Volunteers upon Hypertonic Saline Infusion
Neurophysin−1
Oxytocin
female
male
Neurophysin−1 [pM]
Oxytocin [pg/mL]
Start
End
Timepoint
B
NP−1 and OXT Levels in Healthy Volunteers upon Arginine Infusion
Neurophysin−1
Oxytocin
female
male
Neurophysin−1 [pM]
Oxytocin [pg/mL]
Start
End
Timepoint
C
NP−1 and OXT Levels in Healthy Volunteers upon Oral Macimorelin
Neurophysin−1
Oxytocin
female
male
Neurophysin−1 [pM]
Oxytocin [pg/mL]
Start
End
Timepoint
D
NP−1 and OXT Levels in Healthy Volunteers upon Glucagon Injection
Neurophysin−1
Oxytocin
female
male
Neurophysin−1 [pM]
Oxytocin [pg/mL]
0min
60min
120min
180min
Timepoint
