# Supplementary material for: Neurophysin-I dynamics upon different pituitary provocation tests in healthy participants
Source: Endocr Connect. 2026 May 11;15(5):e250929. doi: 10.1530/EC-25-0929 (PMC13188200; doi:10.1530/EC-25-0929)
Supplement: Supplementary file 2 [file supplementary_table.pdf]

Table S1 **The time course and maximum change of plasma NP-1 levels (in pg/mL) in healthy volunteers in response to each provocation test.**

**Neurophysin-I (pg/mL)**				
**Provocation**	**Hypertonic Saline Infusion**	**Arginine Infusion**	**Oral Macimorelin**	**Glucagon Injection**
**Baseline**	553 [349, 1673]	701 [424, 1092]	949 [566, 1452]	819 [475, 1328]
**Stimulated**	593 ([414, 1506]	679 [493, 1029]	888 [652, 1366]	943 [379, 1608]
Data presented as median [IQR]. Conversion factors: 1 pg/mL ≈ 0.1 pM for neurophysin I.				
